# pXRF Skeletal Measurements as an Assessment Tool for Environmental Exposure to Lajes Field–Derived Contaminants (Terceira Island, Azores, Portugal)

**DOI:** 10.1007/s12011-025-04582-5

**Published:** 2025-03-20

**Authors:** Félix Rodrigues, António Félix Rodrigues, Vítor Matos, Armando Mendes, Maria Teresa Ferreira

**Affiliations:** 1 Department of Life Sciences, Laboratory of Forensic Anthropology, Centre for Functional Ecology, University of Coimbra, Calçada Martim de Freitas, 3000-456 Coimbra, Portugal; 2 Department of Life Sciences, Research Centre for Anthropology and Health, University of Coimbra, Calçada Martim de Freitas, 3000-456 Coimbra, Portugal; 3 Investigation Institute for Agrarian Technologies and Environment, University of the Azores, Rua CapitãO JoãO d’Ávila, São Pedro, 9700-042 Angra do Heroísmo, Portugal; 4 Laboratory of Biological Anthropology, Department of Biology, School of Science and Technology, University of éVora, Évora, Portugal; 5 Centre for Humanistic Studies, University of the Azores, Rua da Mãe de Deus, 9500-321 Ponta Delgada, Portugal

**Keywords:** Environmental contamination, X-ray fluorescence, CEI/Açores, Human skeletal remains

## Abstract

Lajes Field is an Atlantic Portuguese military air base that has been used by the USA since the Cold War, primarily for intercontinental refueling. For this purpose, large fuel tanks and an extensive pipeline network were constructed within the municipality of Praia da Vitória, on Terceira Island, Azores. Over the past two decades, fuel leaks were detected and confirmed to have contaminated soils and the aquifers that supply water for public use. For the latter, identified contaminants include TPH, PAH, BTEX, VOCs, and metals. Although risk assessment reports have identified unacceptable risks to human health, and journalistic investigations suggest unusually high cancer rates, no assessment on possible human exposure has been conducted to date. To address this gap, metals, serving as a *proxy* for overall contamination exposure, were measured using portable X-ray fluorescence (pXRF) in the First Identified Skeletal Collection of the Azores (CEI/Açores). A total of 64 skeletons with known places of last residence were selected (44 from Angra do Heroísmo, where no exposure risk is present, and 20 from Praia da Vitória, where risk is present). No significant differences in mean ages at death were observed between the groups, and sex distribution was similar. Additionally, soil samples from 46 graves were analyzed to assess potential diagenesis. Greater concentrations of Sb, As, Cd, Cr, Au, Mo, Sr, Sn, U, and Zr were found in individuals from Praia da Vitória (*p* < 0.05). Soil measurements, Pearson’s correlation test, and a principal component analysis suggest that the differences in Zr and As levels can be partially attributed to diagenesis. For the remaining metals, the observed differences likely result from other factors, including potential contamination exposure, particularly for Cd, Cr, and Mo. Although this pioneering study contributes to the ongoing discussion on the subject, further research should be conducted both in the CEI/Açores and the living population to further discuss this issue.

## Introduction

**Fig. 1:**
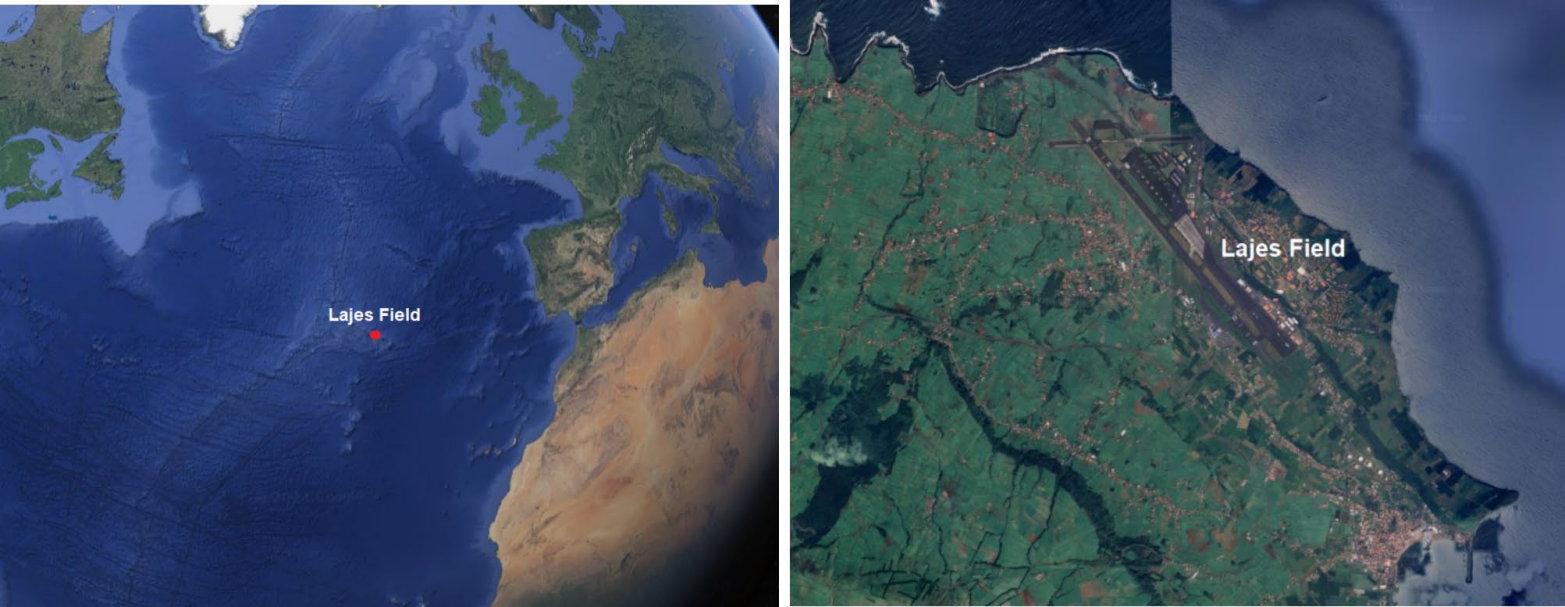
Lajes Field location in the Atlantic and within Terceira. Images adapted from Google Earth

The Lajes Field is a current Atlantic Portuguese air force base, in the Azores archipelago, Portugal (Fig. 1), that counts with a United States of America (USA) air force permanent contingent since 1943 [1]. Although military access to this location had already been granted by the Portuguese to the English during the Second World War, the modern airfield infrastructure was built by the USA after the 1944 bilateral agreement that later led to the inclusion of Portugal in the North Atlantic Treaty Organization (NATO) in 1949 [2].

The geostrategic interest of the USA in this specific location was to establish mid-Atlantic infrastructures capable of supporting long-range communications for its military forces, conducting Soviet submarine searches in the Atlantic [3], projecting military equipment and personnel abroad, and facilitating aircraft intercontinental refueling. Also, the Azores were the last line of US defense in case of a Soviet full invasion of western Europe, which was thought to be imminent [2, 4]. For all these reasons, the USA considered the Azores as the most important geographic point outside its own national territory [4].

The development of large-scale military logistics on a small island also created new job opportunities for locals. Additionally, it allowed the introduction of American technology and media, such as radio, television, and cinema [1]. Besides these economic, cultural, and social dynamics, military operations, such as transatlantic refueling, remained invulnerable. In fact, refueling was one of Lajes Field’s primary purposes during the Cold War, and even though this conflict ended in 1991 with the dissolution of the Warsaw Pact, the base maintained its importance in the subsequent conflicts of the Gulf [5], Afghanistan, and Iraq [6]. Its logistical importance was later reduced due to the budget downsizing in 2012, when the USA relocated resources to the indo-pacific [7]. Nevertheless, Lajes field regained relevance in consequence of today’s Ukrainian war and the middle-east conflict [8].

The initial bilateral agreement between Portugal and the USA, signed in 1944, was intended to expire in 1962. However, as we highlighted, successive periods of international instability, involving directly or indirectly the USA, reinforced Lajes’ strategic importance and led to the extension of the agreements until today [9].

**Fig. 2:**
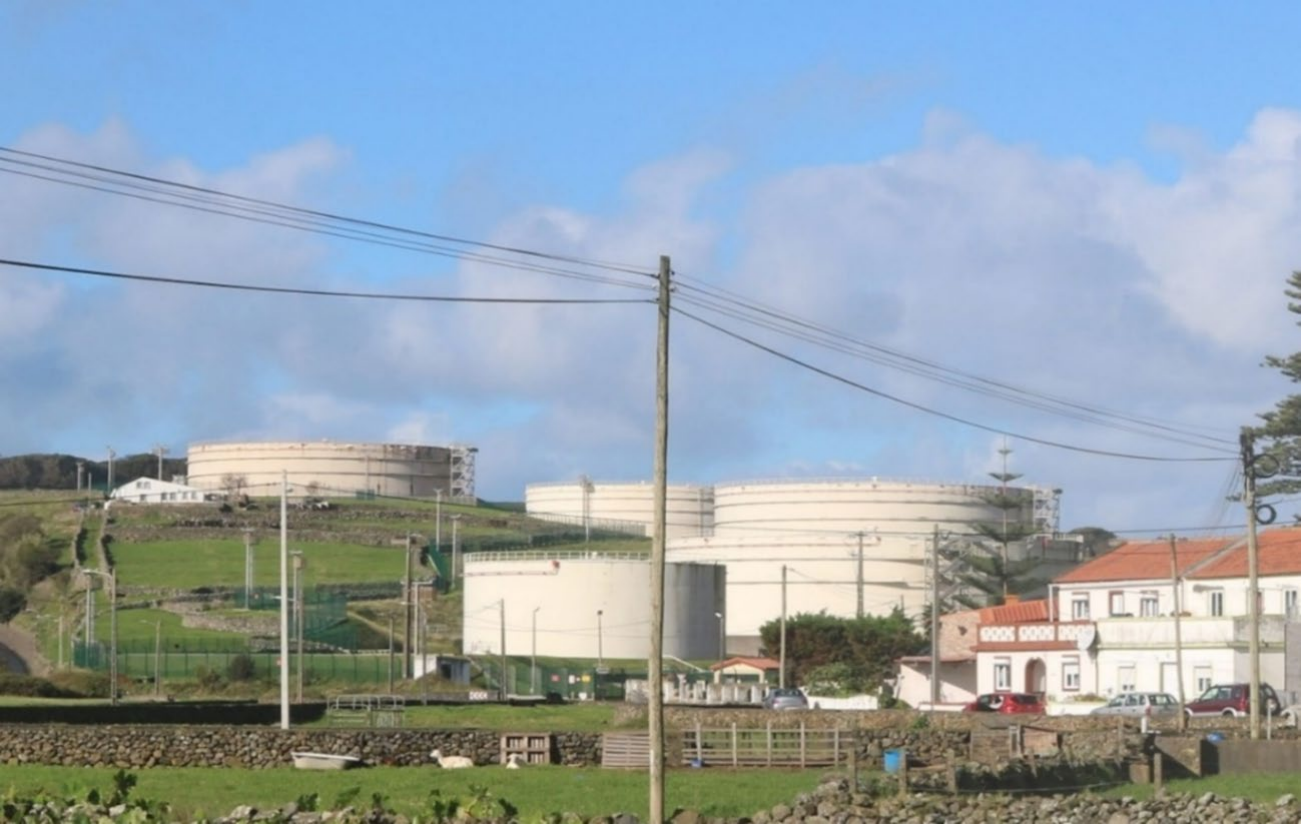
South Tank Farm in Praia da Vitória, Terceira

Since the Northern American presence in Terceira became permanent, facilities capable of sustaining large-scale operations were built. Among them were large tank complexes and the associated pipeline network, capable of storing and flowing hundreds of millions of fuel liters [7]. Considering that major fuel spills were known and several others were suspected in these structures, the North Americans commissioned a study, in 2003, known as the *DISCO study* (Discovery of Suspected and Contaminated Sites Study) [10]. The study was conducted by the private company *CH2MHILL*, with the aim of assessing soil and groundwater quality. Some of the principal locations to study were the South Tank Farm (Fig. 2), Cinder Pit, Military Highway Spill, and the Main Gate.

The resulting report, as well as another from 2005 [11] authored by the same company, identified numerous sites contaminated with hydrocarbon derivatives, highlighting potential risks to human health.

Studies on human health risks from exposure to these contaminants in some of these sites were also performed by *Bathe Environmental Associates*, highlighting unacceptable risks to develop both cancerous and non-cancerous diseases [12, 13].

In the years that followed, the 65th Air Base Wing command (USA) continued the investigations through other independent private companies such as AMEC [14, 15]. These investigations persistently detected hydrocarbon-derived contaminants in several military sites, affecting both soil and the aquifers from which groundwater is collected for public distribution in Praia da Vitória Municipality.

Nonetheless, all the reports mentioned above only became public in 2008 [16]. While technically and scientifically coherent, their publication led to public and political engagement, prompting the Regional Government of the Azores to initiate its own studies, which were first delegated to the National Laboratory of Civil Engineering (LNEC) and later also to the University of The Azores [17].

Several LNEC studies, conducted between 2010 and 2018 [18–21] as well as one from the University of the Azores in 2017 [17], detected the same contaminants, but their concentrations, environmental distributions, and the results interpretations differed from their American and Portuguese peers [21]. Nevertheless, the IITA report (University of the Azores) still emphasized the high vulnerability of major public water extraction wells to contamination, as well as unacceptable risk for human exposure.

It was only in the most recent LNEC report, in 2020 [20], that interpretations became unanimous between groups: The environmental contamination is present at various sites within Praia da Vitória municipality, affecting groundwater, and posing risks to human health within and beyond the military perimeter.

The specific contaminants of concern identified through the years of investigations were total petroleum hydrocarbons (TPH), polyaromatic hydrocarbons (PAH), volatile organic compounds (VOCS), BTEX (benzene, toluene, ethylbenzene, and xylene), and metals/metalloids.

Once in scientific consensus, the 45th USA/Portugal Bilateral Commission issued a joint statement acknowledging the nature of the contamination, its potential risks to human health, and the need for cleanup [22]. As previously mentioned, exposure to such contaminants could result in a range of pathologies, including various types of cancer. This is observed in several populations living in regions with similar contaminants [23–27]. In fact, Portuguese national statistics highlight that Praia da Vitória is among the Portuguese municipalities with the highest cancer mortality rates, accounting for 36.8% of all causes of death. This indicates that more than 1 out of 3 deaths in Praia da Vitória are due to cancer. This contrasts with the neighboring municipality of Angra do Heroísmo, where cancer accounts for 26.3% of all causes of death [28]. The latter is closer to the overall national values, which stand at 22.1% [28]. It is important to note that Angra do Heroísmo does not present a risk of contamination exposure. Moreover, both national and local journalistic investigations have shed light on unusual cancer rates in Praia da Vitória, suggesting a potential correlation with the geographical risk of contaminant exposure [29–31].

Despite extensive investigation of this issue over the past decades, involving contaminant identification and monitoring, health risk assessment, and journalistic works covering disease rates by geographical risk of exposure, no study on actual exposure itself has been conducted to date. This assessment is crucial either for preventing additional disease burden in individuals and the community, implementing protective measures, and integrating contaminant exposure into medical differential diagnoses or, on the other hand, if no evidence of exposure is found, to ensure a minimum level of social tranquility.

In this paper, we aim to determine, for the first time, whether the local population has been exposed to these contaminants through the First Identified Skeletal Collection of the Azores (CEI/Açores). Since this collection comprises skeletal remains of individuals who had their last residence in Terceira Island, both at-risk and risk-free locations, and considering the human bone as an *antemortem* bio-reservoir for metals and metalloids [32, 33], we measured their concentrations, serving as a proxy for overall contamination exposure.

The selected technology, portable X-ray fluorescence (pXRF), has recently been applied to identified skeletal collections to measure metal concentrations in human bones. These studies have successfully assessed these concentrations and their differences according to various pathologies [34–36]. For this reason, if differences in metal concentrations exist between the populations of Angra do Heroísmo and Praia da Vitória as a result of environmental contamination exposure, they should be detectable by this technology.

## Sample and Methods

### The First Identified Skeletal Collection of the Azores (CEI/Açores)

The First Identified Skeletal Collection of the Azores (CEI/Açores), located in Praia da Vitória, Terceira Island, is under the tutelage of the Municipality. Curated by the first author, an anthropology researcher at the University of Coimbra, it was assembled in 2023 to provide a dignified outcome for abandoned cemetery remains while enabling research. As the curator is solely responsible for the research strategies, no additional permissions were required for this study.

The CEI/Açores comprises 75 skeletons, each accompanied by information regarding age at death, year of death, sex, and place of last residence. Additionally, details about the cemetery and grave from which they were exhumed are available, as well as soil samples from 46 graves that were collected at the time of exhumation, approximately 15 cm above the coffin.

All skeletons were meticulously cleaned with distilled water and properly dried at room temperature prior to any skeletal measurements.

The selection criteria for the skeletons to measure were based on the place of last residence and year of death. Only individuals who had their last residence in Santa Cruz, Praia da Vitória (location presenting a risk of exposure), and in Angra do Heroísmo (location without risk of exposure) were selected. Additionally, individuals with deaths occurring before the year 2000 were excluded, as detailed technical reports of contamination are dated after this year.

A total of 64 skeletons were selected based on defined criteria. Of these, 44 had their last residence in Angra do Heroísmo, while the remaining 20 were in Praia da Vitória. These individuals were born between 1922 and 1984, and their deaths occurred between 2005 and 2013. The sex proportion was similar between groups, namely 23 masculine individuals (52%) and 21 feminine individuals (48%) in Angra do Heroísmo, while 12 masculine (60%) and 8 feminine (40%) individuals were in Praia da Vitória. A chi-square goodness of fit test was applied to ensure this similarity between sexes (*p* = 0.655 in Angra do Heroísmo and *p* = 0.491 in Praia da Vitória). Table 1 presents sex and age distributions between locals.

**Table 1:** Distribution of the selected skeletons by sex and age at death group

	Angra do Heroísmo			Praia da Vitória		
Age (years)	< 50	> 50	Total	< 50	> 50	Total
Masculine	4	19	23	1	11	12
Feminine	2	19	21	1	7	8
Total	6	38	44	2	18	20

Mean ages at death were 70.6 ± 14.1 years in Angra do Heroísmo and 72.1 ± 14.5 years in Praia da Vitória. Statistical ages at death differences were tested using Student’s *t*-test. No significant differences between groups (*p* = 0.2) were observed.

### The pXRF Measurements

For metal and metalloid concentration measurements in the skeletons, we utilized Hitachi pXRF XMET 8000 Expert Geo. The instrument is equipped with an X-ray tube functioning at a maximum of 50 kV of voltage and 200 µA of current, and it is capable of measuring concentrations of elements in a sample ranging from the atomic number of magnesium (Mg) to uranium (U) [37]. Its accuracy was tested with SiO_2_ and BAM-U110 samples prior to every set of measurements. Nevertheless, it was not possible to determine a relative technical error of measurement using a reference material, as bone ash with a known composition, as recommended by Gomes et al. (2024), could not be obtained [38].

**Fig. 3:**
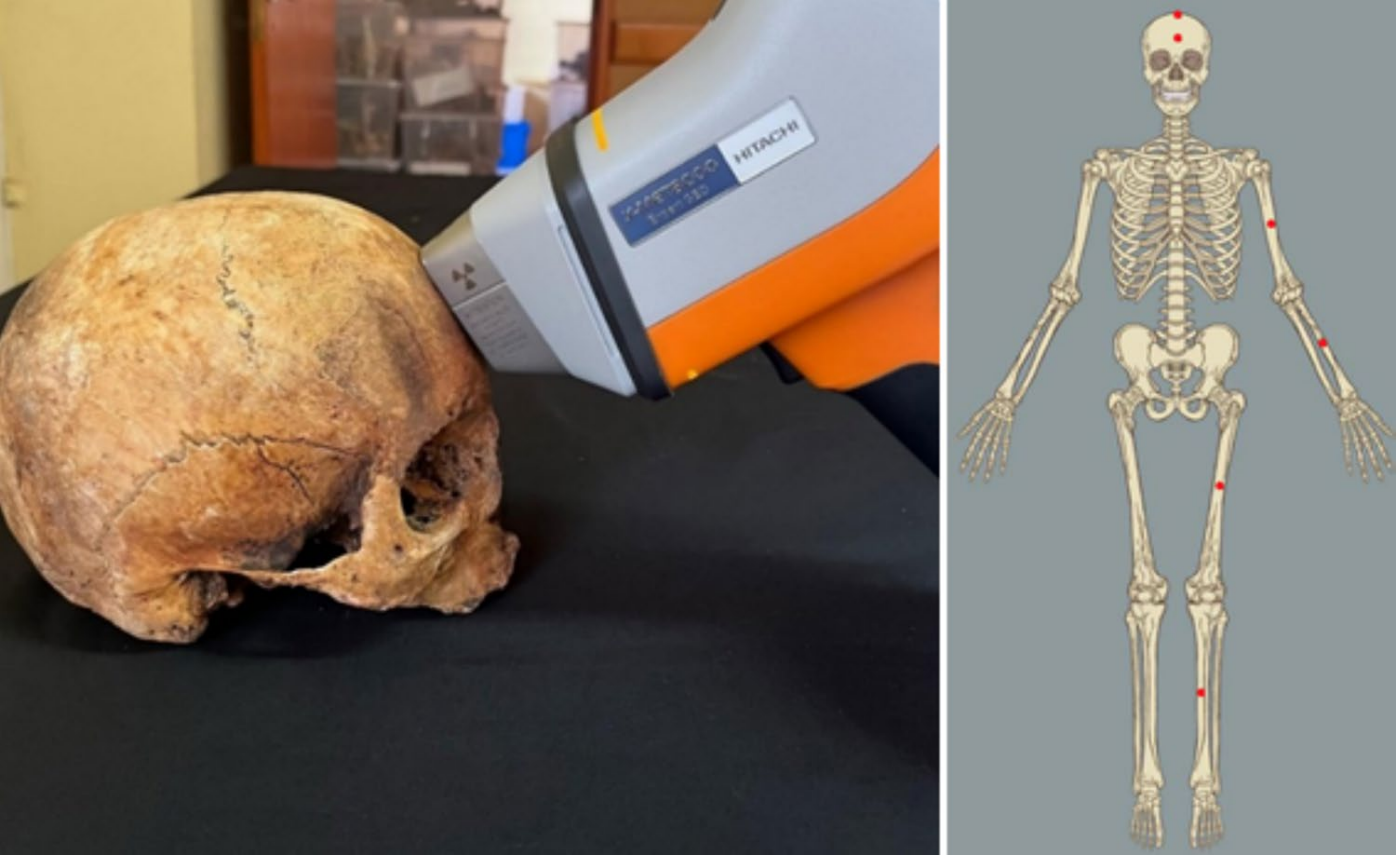
PXRF measurements in a frontal bone from the CEI/Açores on the left and the visual representation of all measures taken, in red dots, on the right (frontal, bregma, midshafts of left humerus, radius, tibia, and femur)

Figure 3 illustrates the measuring process in a human skull from the CEI/Açores and a representation of all the measures taken in each skeleton.

The selected pXRF mode was CN-Soil, and the results show as the mean of two measures (each with 30 s of acquisition time).

For this study, and considering possible concentration variability between bones, six skeletal locations were measured for each individual, specifically the frontal bone, the bregma, and the midshafts of the left humerus, radius, tibia, and femur (Fig. 3), while avoiding bone lesions, damage, and stains, in compliance with Gomes et al.’s (2024) guidelines [38]. Following the same recommendations, the concentrations for each individual were calculated as a simple mean of all measurements. Once all means were calculated, the individuals were grouped based on their place of last residence, and statistical comparisons were conducted. As occasional outliers were observed in the dataset, along with violations of normality (Shapiro–Wilk test) and differences in variance (Levene’s test), the comparison between groups was conducted using a Mann–Whitney *U* test. This non-parametric test is the most adequate in such cases [39].

In order to test for diagenesis, as recommended by Gomes et al. (2021) [35], the 46 available grave soil samples were individually homogenized and analyzed using the same pXRF specifications. Soil results were compared between locations, also using the Mann–Whitney *U* test.

Additionally, Pearson’s correlation was applied to assess the relationship between soil metal concentrations and those found in the skeletons.

At last, a principal component analysis (PCA), considering eigenvalues and varimax orthogonal rotation, was conducted to further interpret bone concentration variance. By grouping metals based on their variance and visualizing their distribution along the two principal component axis, potential sources of metals can be hypothesized (e.g., environmental contamination or other factors). All statistical analyses were conducted in JASP 0.16.00 statistical software, and graphics were generated in Python.

## Results

Descriptive statistics for the detected elements in the skeletal samples are presented by location of last residency in Table 2, namely for antimony (Sb), arsenic (As), barium (Ba), cadmium (Cd), calcium (Ca), chromium (Cr), copper (Cu), gold (Au), iron (Fe), lead (Pb), manganese (Mn), mercury (Hg), molybdenum (Mo), nickel (Ni), silver (Ag), strontium (Sr), tin (Sn), titanium (Ti), uranium (U), zinc (Zn), and zirconium (Zr). The results of the Mann–Whitney *U* test, used to evaluate differences between Angra do Heroísmo and Praia da Vitória populations, are also presented in Table 2. For metals where differences exist, the population with the higher means is the one with significantly greater concentration (*p* < 0.05).

**Table 2:** Metal concentrations in a skeletal sample from the CEI/Açores

	Skeletons from Angra do Heroísmo (No risk; *n* = 44)		Skeletons from Praia da Vitória (Risk is present; *n* = 20)		
Element	Mean + SD (ppm)	Min–max (ppm)	Mean + SD (ppm)	Min–max (ppm)	*p*-values
Antimony (Sb)	19.2 ± 3.9	7.3–26.3	23.0 ± 3.0	15.1–27.0	< 0.001
Arsenic (As)	0.9 ± 0.6	< LOD–3.0	1.3 ± 0.9	< LOD–3.7	0.019
Barium (Ba)	146.7 ± 31.3	69.2–215.6	149.2 ± 29.2	101.9–206.5	0.353
Cadmium (Cd)	10.2 ± 3.7	2.8–7.7	12.7 ± 3.1	7.7–18.8	**0.005**
Calcium (Ca)*	251 ± 21.3	180–285	257 ± 18.3	220–284	0.123
Chromium (Cr)	8.2 ± 14.0	< LOD–89.3	24.62 ± 34.8	< LOD −146	**0.025**
Copper (Cu)	7.1 ± 5.5	< LOD–25	12.8 ± 28.9	< LOD–134.4	0.490
Gold (Au)	23.4 ± 4.9	9–29.7	26.5 ± 4.8	14.8–32.8	**0.003**
Iron (Fe)*	16 ± 8.4	6–46.1	26.3 ± 2.7	8.9–91.7	0.310
Lead (Pb)	12.2 ± 5.4	3.6–22.6	13.9 ± 8.4	5.7–37.8	0.360
Manganese (Mn)	335 ± 246.7	22.4–1033	290.5 ± 159	95.8–614	0.771
Mercury (Hg)	7.5 ± 2.7	3.3–15.5	8.7 ± 3.4	4–14.7	0.110
Molybdenum (Mo)	4.5 ± 1.0	1–6.8	5.3 ± 1.3	4–10.6	**0.006**
Nickel (Ni)	17.8 ± 5.8	8.2–35.6	17.8 ± 5	10.3–30.3	0.400
Silver (Ag)	4.3 ± 4.5	< LOD–22.4	5.3 ± 3.4	< LOD–12.7	0.063
Strontium (Sr)	120 ± 33.7	71.7–240	150 ± 42	84.2–223.5	**0.004**
Tin (Sn)	53.2 ± 8.1	29.4–67.0	62.3 ± 9.4	37.4–76.1	** < 0.001**
Titanium (Ti)	108.8 ± 98.6	< LOD–421	57.9 ± 66.5	< LOD–228	0.900
Uranium (U)	1.2 ± 0.6	< LOD–2.3	2.3 ± 1.3	< LOD–9	**0.001**
Zinc (Zn)	879.9 ± 472.8	341–2658	739.4 ± 350	274–1567	0.890
Zirconium (Zr)	4.1 ± 5.9	1–39.4	3.9 ± 2.1	< LOD–10.7	**0.040**

Bold highlights values with statistical significance; < *LOD*, below the limit of detection

To address the exceptionally large values of Ca and Fe in Table 2, Ca results were divided by a factor of 1000 and Fe by 100. Both were marked with an asterisk (*).

As shown in Table 2, statistically greater concentrations were found in Praia da Vitória skeletons for Sb, As, Cd, Cr, Au, Mo, Sr, Sn, U, and Zr.

In order to determine if these differences reflect exposure to overall contamination related to Lajes Field, factors such as diagenesis need to be excluded. Therefore, two analyses were conducted: measure and compare grave soil concentrations of significant metals by location (Table 3) and Pearson’s correlation to assess the relation between grave soil and bone concentrations (Table 4).

**Table 3:** PXRF grave soil results for the significant metals

	Soil samples from Angra do Heroísmo Graves (*n* = 31)		Soil samples from Praia da Vitória graves (*n* = 15)		
Element	Mean + SD (ppm)	Min–max (ppm)	Mean + SD (ppm)	Min–max (ppm)	*p*-values
Antimony (Sb)	19.7 ± 8.1	5–36	18 ± 10.7	6–48	0.849
Arsenic (As)	5.6 ± 3.4	< LOD–14	8.8 ± 3.3	3–16	**0.002**
Cadmium (Cd)	11.3 ± 7.3	< LOD–32	12.7 ± 6.3	6–28	0.255
Chromium (Cr)	28.9 ± 8.4	17–53	24.5 ± 5.7	13–35	0.938
Gold (Au)	24.6 ± 7.9	10–40	24.8 ± 9.2	< LOD–35	0.328
Molybdenum (Mo)	7 ± 3.3	2–13	10.3 ± 3.3	5–15	**0.002**
Strontium (Sr)	95.6 ± 28.7	48–178	98.6 ± 27.1	52–153	0.300
Tin (Sn)	75.7 ± 13.7	51–102	68.8 ± 9.8	47–82	0.974
Uranium (U)	1.3 ± 1.4	< LOD–6	2.1 ± 2.2	< LOD–8	0.113
Zirconium (Zr)*	103 ± 23.2	69–143	136 ± 15.7	107–171	** < 0.001**

Bold highlights values with statistical significance; < *LOD*, below the limit of detection

**Table 4:** Pearson’s correlation between bone and respective grave soil metal concentrations

Element	Pearson’s *r*	*p*-values
Antimony (Sb)	− 0.18	0.364
Arsenic (As)	0.41	**0.029**
Cadmium (Cd)	0.37	**0.040**
Chromium (Cr)	− 0.14	0.460
Gold (Au)	− 0.02	0.910
Molybdenum (Mo)	0.11	0.560
Strontium (Sr)	− 0.20	0.300
Tin (Sn)	− 0.24	0.210
Uranium (U)	0.12	0.540
Zirconium (Zr)	0.40	**0.048**

Bold highlights values with statistical significance.

Table 3 indicates grave soil concentrations in Angra do Heroísmo and Praia da Vitória, and also the Mann–Whitney *U p*-values. In cases where significant differences exist, the location with the higher means is the one with significantly greater concentration. Similarly, to Table 2, in order to account for the exceptionally large values of Zr, these were divided by a factor of 10 and are marked with an asterisk (*).

Among the evaluated metals, Praia da Vitória grave soils presented significantly greater concentrations for As, Mo, and Zr, which suggest their higher concentrations in the skeletal samples could be related to diagenesis.

Significant correlations were also observed between soil and bone concentrations for As, Cd, and Zr (Table 4).

No relation was found between soil and bone concentrations of Mo even though its concentration is greater in Praia da Vitória grave soils.

Only As and Zr showed differences both in skeletons and in soils while also having a significant Pearson’s correlation between them.

In order to further interpret the results, a PCA was applied for the significant bone metals.

The PCA identified three principal components that explain 94% of the total variance of the analyzed metals and metalloids in the skeletons.

**Fig. 4:**
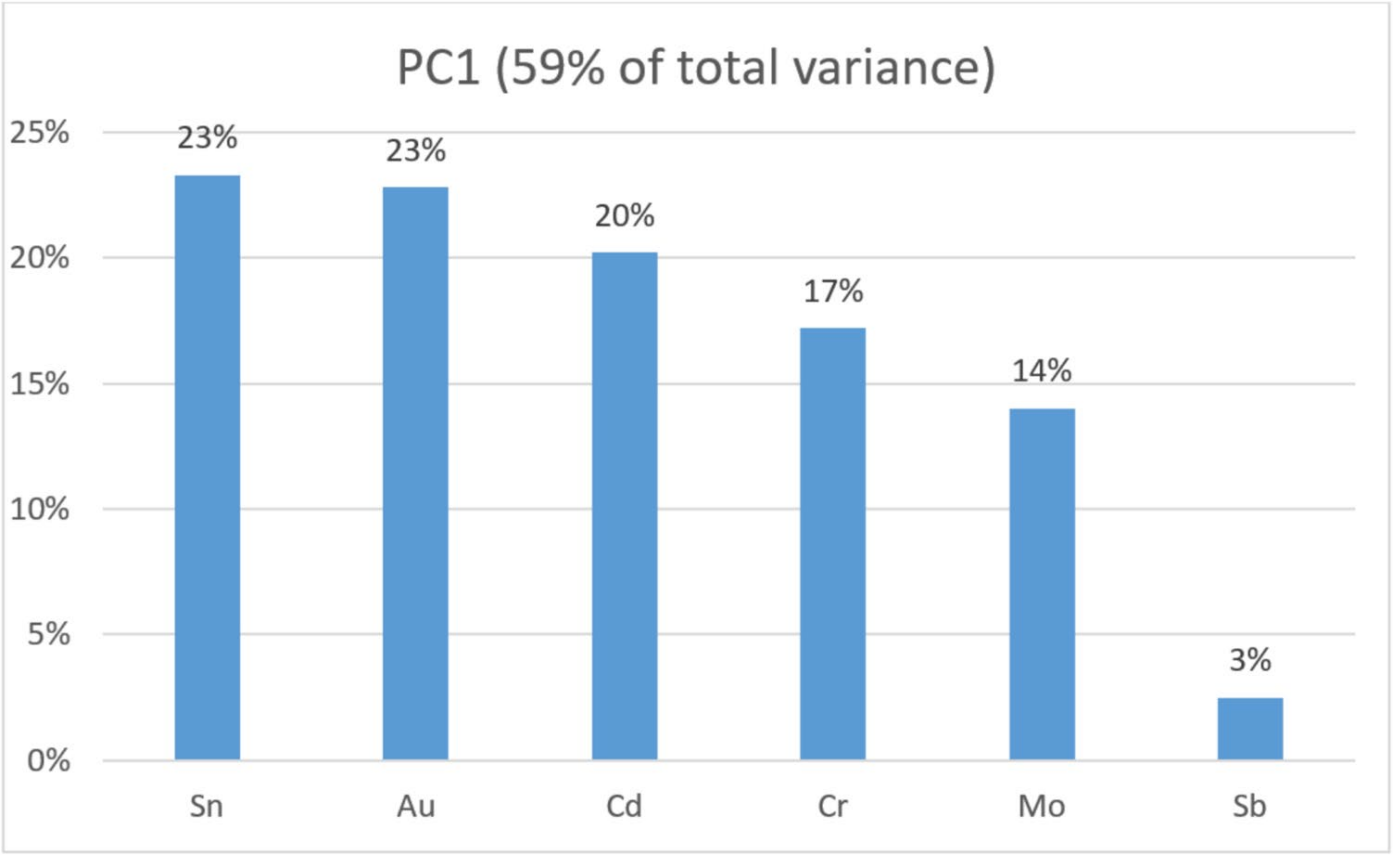
Individual contribution of each metal to the total variance in PC1

**Fig. 5:**
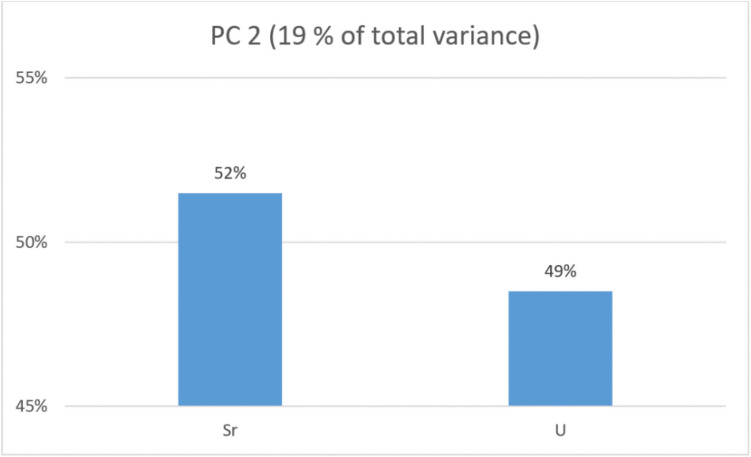
Individual contribution of each metal to the total variance in PC2

**Fig. 6:**
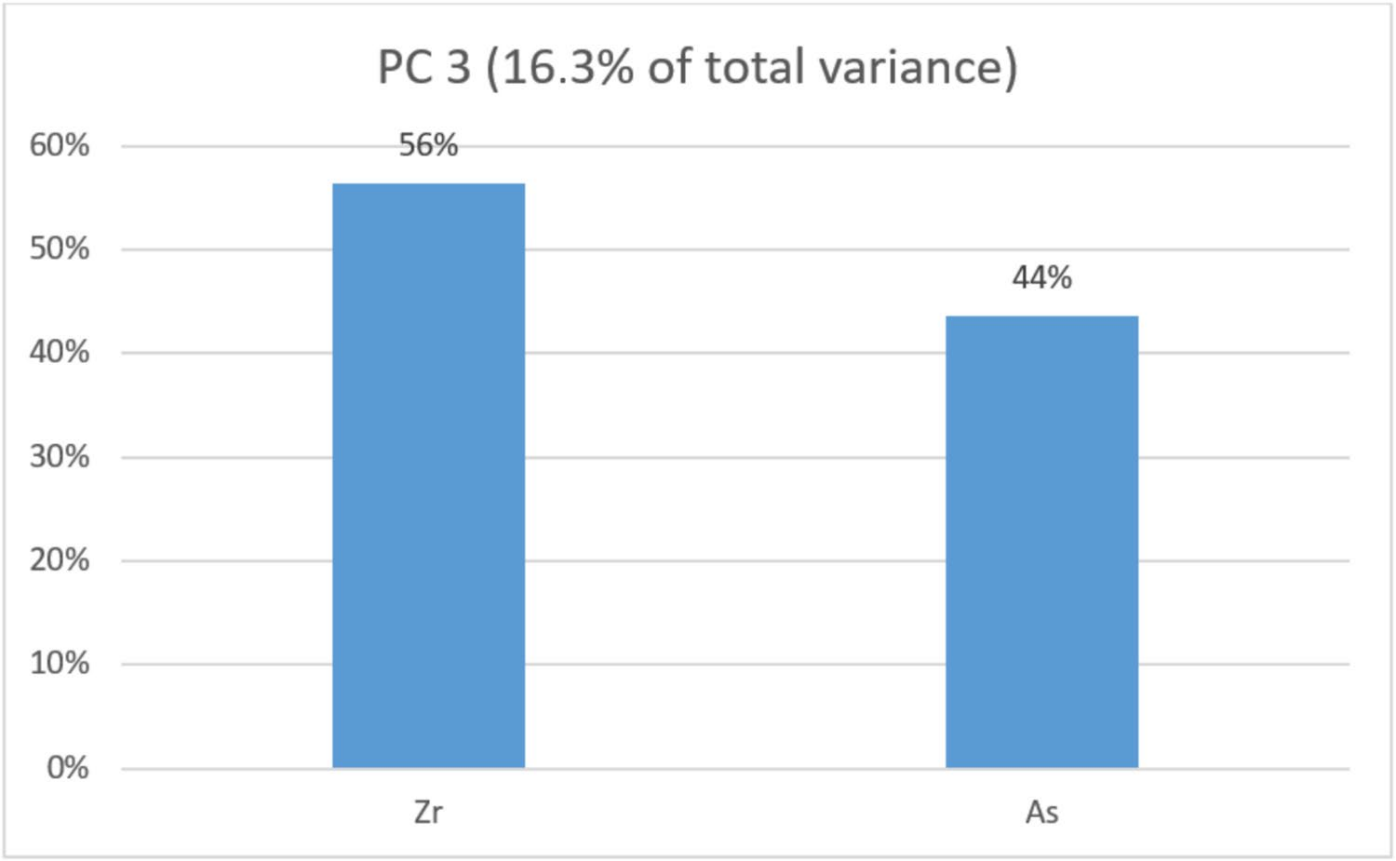
Individual contribution of each metal to the total variance in PC3

**Fig. 7:**
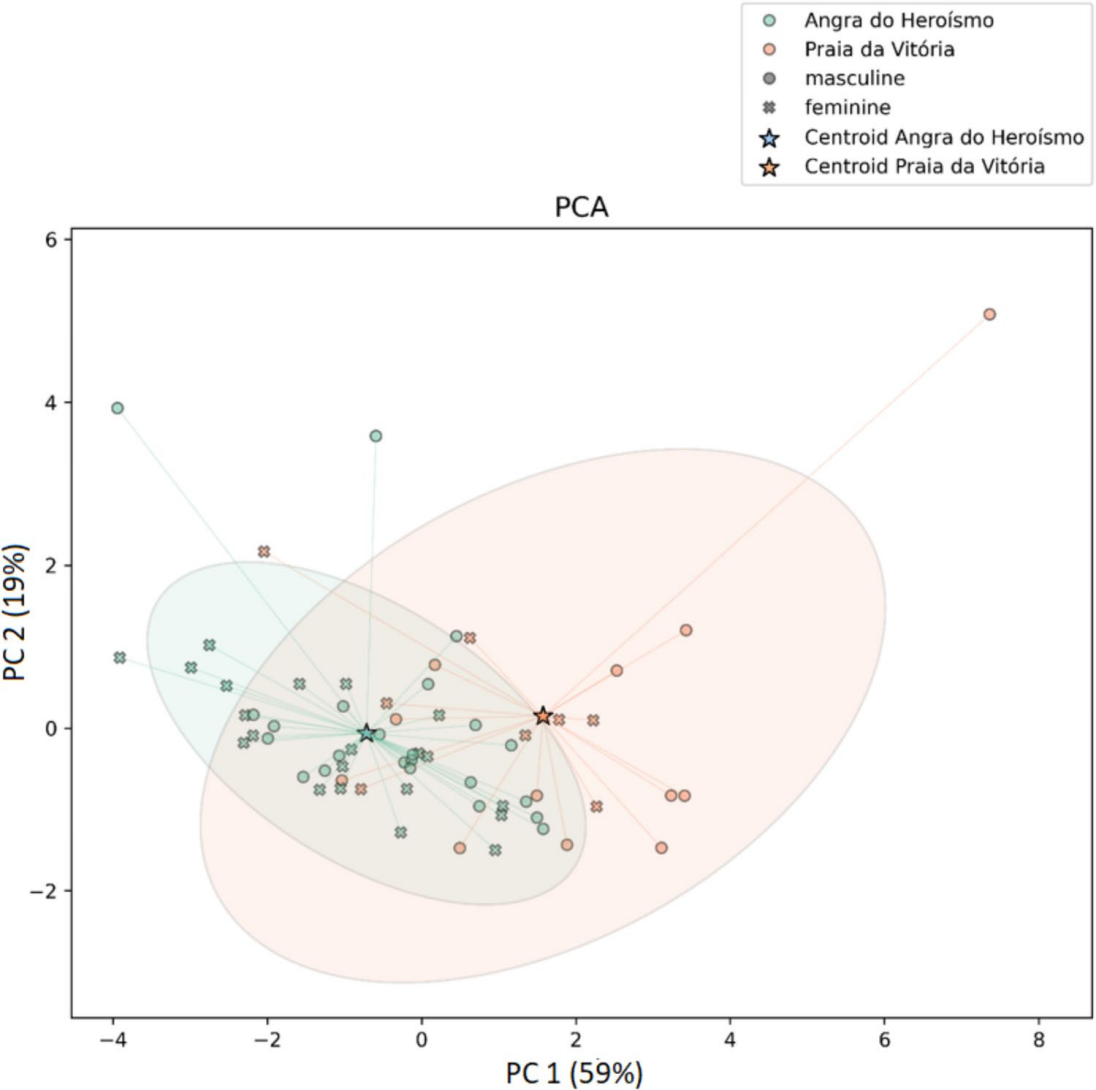
Visual representation of the distribution of each individual along the two principal component axis

The first (PC1) that grouped Sn, Au, Cd, Mo, Cr, and Sb explains 59% of the total variance. The second (PC2) groups Sr and U and explains 19%. The third (PC3) groups Zr and As and explains 16.3% of the total variance. The contributions of each metal to their respective principal components are presented as percentages in Figs. 4, 5, and 6. Figure 7 presents the distribution of each individual along the two principal component axis.

For PC1, Sn, Au, Cd, Cr, and Mo account for the majority of the total variance, displaying similar percentage weights, while the contribution of Sb is minimal.

PC2 groups Sr and U, which contribute almost equally to the total variance.

In PC3, Zr and As are the only metals observed, contributing similarly to the total variance.

In Fig. 7, two distinct location clusters—Angra do Heroísmo and Praia da Vitória—with 95% confidence ellipses are observed as well as the respective centroids.

## Discussion

The utilized skeletal sample, and the available biographic information about their individuals, enabled a first attempt to test the differential exposure hypothesis between two locations in Terceira Island.

The obtained pXRF measurement results showed differences with statistical significance in a vast array of metals between groups, being greater in Praia da Vitória skeletons. It is relevant to highlight that a part of them were identified in monitoring reports as environmental contaminants stemming from military structures in this municipality. This is the case of As, Cd, Cr, and Mo [20].

In the case of the remaining significant metals, Sb was systematically reported beyond detection limits in the same reports [19, 20] and the remaining metals (Au, Sr, Sn, U, and Zr) were never tested either in water or soil samples, and therefore their environmental concentrations in Terceira island remain unknown.

The additional grave soil sample analysis was also relevant to test the diagenesis hypothesis, as soil can alter bone chemical composition [40]. Indeed, Praia da Vitória grave soils showed higher concentrations of Mo, As, and Zr, but only the latter two exhibited a significant Pearson’s correlation with the skeletal concentrations. This strongly suggests that differences in skeletal samples for As and Zr are partly due to diagenesis, whereas the remaining metals are better explained by other factors, including possible contamination exposure during life.

The PCA, which is an exploratory analysis on metal variance, helps to further interpret our results and hypothesize sources.

PC1, which accounts for the highest metal variance in the skeletons, grouped Sn, Au, Cd, Cr, Mo, and Sb. With the exception of Sb, the percentage contributions of each metal to this component are similar (Fig. 4). While smoking can significantly influence Cd concentrations in humans [41, 42], its association and proportionality with Mo and Cr are better explained by other factors. Smokers also tend to have higher Pb concentrations [41], but no significant differences were observed between Angra do Heroísmo and Praia da Vitória for this metal. Additionally, Cd, Cr, and Mo are known contaminants in Praia da Vitória, as already stated. This further suggests environmental exposure as a possible source for the greater levels of these metals in Praia da Vitória skeletons.

In PC2, the association of Sr and U (Fig. 5) could reflect natural background levels acquired through ingestion, especially water [43]. Although no background environmental concentrations for these metals are known, besides their levels in the graves, which are similar between cemeteries and inferior to those observed in the bones, isotopic analysis should be implemented in the future to further elucidate about the possible source of exposure. In other words, isotopic analysis would clarify whether the different concentrations in the bones reflect different natural levels between Angra do Heroísmo and Praia da Vitória or if there is an unknown anthropogenic source causing significant differences in bone concentrations.

In PC3, the association of Zr and As aligns with previously obtained results and could represent diagenesis. As this is the most probable case, the variances in PC1 and PC2 should be influenced by sources other than bone-soil diagenesis, as discussed above.

Regarding the visual distribution of each individual along the two principal component axis (Fig. 7), partial overlap is observed between the ellipses of both locations, although the respective centroids suggest some separation between groups. This visualization shows the individual variability, which results from a complex interplay between humans and the environment. Factors such as the duration and route of exposure, as well as individual metabolism and metal excretion capacity [44], likely contribute to this variability. In one example case from Praia da Vitória, the individual’s last known residence was a nursing home (Lar Dom Pedro V). This particular case, visualized in the overlapping area of the ellipses, raises questions about whether he lived in Praia da Vitória for his entire life or only during the final stage. If the latter, it remains uncertain whether the duration of residence in Praia da Vitória was sufficient to increase metal concentrations in his bones compared to the levels he had before. The same question applies to all individuals in the study regarding the place of last residence.

Individual variability could also justify the standard deviation of Cr in the skeletons being greater than the mean. The presence of Cr outliers in individuals from both locations highlights the complexity of individual variance. However, outliers are expected in contamination contexts for the reasons mentioned above: an individual may have greater exposure to contaminants or a lower biological capacity for their excretion. For instance, significant levels of Cr were observed in the urine of children living in Cr contaminated sites in New Jersey in the early 90 s, with the standard deviation being greater than the mean due to the presence of outliers [45].

Even so, in the present study, significant differences between locations are observed using the Mann–Whitney *U* test. This test minimizes the influence of outliers while still revealing significantly different concentrations between groups. These differences may be attributed to higher contamination exposure in Praia da Vitória residents.

It is also proven that chronic exposure to metals can cause a vast array of diseases, involving multiple organ systems [46], in which the skeleton is included [47]. Therefore, further anthropological investigations should be conducted on the identified skeletal collection of the Azores to determine if metal concentrations are related to specific bone disease rates and also to the geographical location of last residence.

As previously stated, the sample size in this study was limited, with more individuals from Angra do Heroísmo than from Praia da Vitória, but this difference reflects the proportions of the living populations of each municipality. Angra do Heroísmo has twice the population of Praia da Vitória, which ultimately affects the number of exhumations in each cemetery and consequently the available number of skeletal remains to study. Nevertheless, as the CEI/Açores is currently being enlarged and is expected to grow to 200 individuals in the next year, the results of this study should be updated with a greater sample and include the remaining advice in the pXRF guidelines for human bone analysis [38]. A larger sample will also allow tests on the variability between bones of the same individual, as well as differences by sex and age, potentially enabling additional discussions.

Even though the human skeleton can provide valuable clues regarding exposure to contaminants stemming from Lajes Field, and possible health consequences resulting from exposure, ultimately it is the living populations that must be protected from present day risks. For this reason, additional health-related studies for the living populations are of utmost importance. Only these can elucidate about exposure and possible health-related burdens in the present. Also, the living can provide far more detailed biographic information than the dead, for instance, the street they live in, their professions, timelines, past and present illnesses, their major source of water and food consumption (tap or bottled water) and their medications. This information could then be crossed with concentrations in the urine or blood not only for metals [48–51] but for the remaining contaminants as well [52–54], therefore enabling a broader comprehension of the issue. They could also elucidate about the impact of risk perception among locals and respective measures they adopted to prevent exposure since this subject became known to the public.

## Conclusions

Considering the decade-long environmental contamination studies at Lajes Field and Praia da Vitória, the work conducted on the First Identified Skeletal Collection (CEI/Açores) is the first to test if contaminant exposure had occurred in the local population.

Through the use of pXRF, this work identified statistically significant differences in the concentrations of various metals and metalloids between individuals who lived in Praia da Vitória, an area with known environmental contamination risks linked to the Lajes Field military base, and Angra do Heroísmo, a city approximately 23 km away where no such risk is expected.

The findings highlight greater concentrations of Sb, As, Cd, Cr, Au, Mo, Sr, Sn, U, and Zr in the skeletal remains of individuals from Praia da Vitória.

Further analysis, including the assessment of grave soil samples, Pearson’s correlation, and a PCA, suggested that As and Zr concentrations in the bones could partly be attributed to soil diagenesis since these skeletons were previously buried in local cemeteries. However, for other metals, such as Cd, Cr, and Mo, the greater concentrations in skeletal samples could possibly reflect contamination exposure from environmental sources linked to military activities.

Although this pioneering study contributes to the ongoing discussion on the subject and suggests possible future research strategies, only comprehensive approaches can further elucidate about the complex interplay between environment and local livelihood, both in the past and the present.

## Data Availability

No datasets were generated or analysed during the current study.
